# Modified Release of the Pineal Hormone Melatonin from Matrix Tablets Containing Poly(*L*-lactic Acid) and Its PLA-*co*-PEAd and PLA-*co*-PBAd Copolymers

**DOI:** 10.3390/polym14081504

**Published:** 2022-04-07

**Authors:** Marilena Vlachou, Angeliki Siamidi, Dionysia Anagnostopoulou, Evi Christodoulou, Nikolaos D. Bikiaris

**Affiliations:** 1 Division of Pharmaceutical Technology, Department of Pharmacy, School of Health Sciences, National and Kapodistrian University of Athens, Panepistimioupoli-Zografou, 15784 Athens, Greece; asiamidi@pharm.uoa.gr (A.S.); dionanag@pharm.uoa.gr (D.A.); 2 Laboratory of Polymer Chemistry and Technology, Department of Chemistry, Aristotle University of Thessaloniki, 54124 Thessaloniki, Greece; evicius@gmail.com (E.C.); nbikiaris@gmail.com (N.D.B.)

**Keywords:** melatonin, poly(*L*-lactic acid) (PLA), PLA-*co*-PEAd and PLA-*co*-PBAd copolymers, dissolution, modified-release matrix tablets

## Abstract

In terms of drug delivery, the attractive properties of poly(*L*-lactic acid) (PLA) and its aliphatic polyesters, poly(ethylene adipate) (PEAd) and poly(butylene adipate) (PBAd), render them ideal co-formulants for the preparation of modified-release pharmaceutical formulations. Furthermore, we have previously demonstrated that by adding a “softer” aliphatic polyester onto the macromolecular chain of PLA, i.e., PEAd or PBAd, resulting in the formation of the PLA’s copolymers (PLA-*co*-PEAd and PLA-*co*-PBAd, in 95/5, 90/10, 75/25 and 50/50 weight ratios), the hydrolysis rate is also severely affected, leading to improved dissolution rates of the active pharmaceutical ingredients (API). In the present report, we communicate our findings on the in vitro modified release of the chronobiotic hormone melatonin (MLT), in aqueous media (pH 1.2 and 6.8), from poly(*L*-lactic acid) and the aforementioned copolymer matrix tablets, enriched with commonly used biopolymers, such as hydroxypropylmethylcellulose (HPMC K15), lactose monohydrate, and sodium alginate. It was found that, depending on the composition and the relevant content of these excipients in the matrix tablets, the release of MLT satisfied the sought targets for fast sleep onset and sleep maintenance. These findings constitute a useful background for pursuing relevant in vivo studies on melatonin in the future.

## 1. Introduction

Melatonin (*N*-acetyl-5-methoxytryptamine, MLT), the pineal hormone released at night [1,2], regulates the start of sleep in animals, including humans. It has been demonstrated to have hypnotic properties in both animals and humans [3], and has been used to help restore circadian rhythms that have been disarrayed by jet lag, shift work, or aging [4,5]. Melatonin can ameliorate the severity of symptoms and cellular damage caused by SARS-CoV-2 when used as an early adjunct therapy of COVID-19 [6,7], because of its known efficacy as an antioxidant [8,9], anti-inflammatory [10], and immunomodulator [11]. Melatonin’s physiological functions are mediated by a family of G-protein-coupled membrane receptors with a high affinity for melatonin. In mammals, amphibians, and other vertebrates, two receptor subtypes, MT1 and MT2, have been discovered and cloned [12,13], which, when produced in host cells, display the overall pharmacological features of natural melatonin receptors. Recently, high-resolution, room-temperature X-ray free-electron laser (XFEL) structures of MT_1_ and MT_2_ in complex with agonists have revealed melatonin subtype receptor insights into ligand entry and receptor selectivity [14]. Typical examples are Ramelteon^®^, which is a melatonin agonist that is commercially available for the treatment of sleep initiation difficulty and insomnia symptoms [15], and agomelatine (potent melatonin agonist), which is the only currently available antidepressant agent that does not primarily target the monoaminergic system [16]. Tasimelteon is also licensed for the treatment of non-24-h sleep–wake disorder [5].

In recent years, our research group has been working on innovative oral MLT delivery systems for treating sleep onset and sleep maintenance dysfunctions [17,18,19,20]. Because controlled release melatonin administration is known to be more therapeutically helpful in beginning and sustaining sleep than immediate-release in senior insomniacs, we have focused on modified vs. immediate-release tablet formulations. Moreover, the choice for the development of modified-release MLT formulations was based on the hormone’s poor bioavailability and short half-life [20,21,22]. In this report, we communicate our findings on the in vitro modified release of MLT, in aqueous media (pH 1.2 and 6.8), from poly(*L*-lactic acid) and its copolymer matrix tablets, enriched with commonly used biopolymers, such as hydroxypropylmethylcellulose (HPMC K15), lactose monohydrate, and sodium alginate.

Due to its biocompatibility, biodegradability, and excellent physicochemical and mechanical qualities, poly(*L*-lactic acid) (PLA), a commercially accessible synthetic polymer, finds ample use in a variety of applications, also including biomedical applications, such as drug delivery and tissue engineering. PLA can be produced through different polymerization techniques [23], and it comes in the following three different forms, each with its own set of properties: the enantiomers poly(*L*-lactide) (PLLA) and poly(*D*-lactide) (PDLA), as well as the racemate poly(*DL*-lactide) (PDLLA). PLA is an appealing choice for deployment because of its versatility and processability, particularly in the field of pharmaceutical technology [24,25,26,27]. As in the case of other aliphatic polyesters, improved performance and tailored properties (e.g., crystallinity, mechanical performance, and degradation rates) can be achieved indirectly by the synthesis of copolymers.

**Figure 1:**
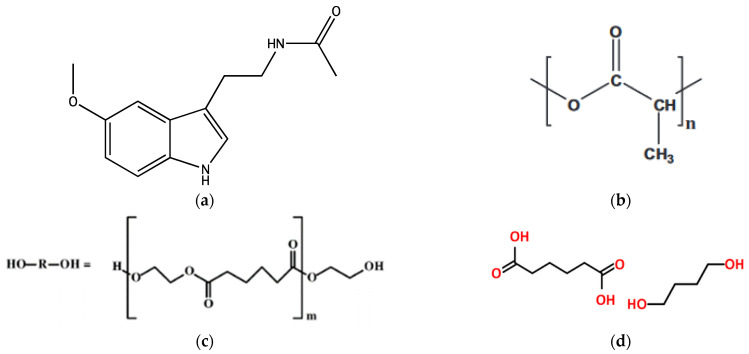
Chemical structures of (**a**) melatonin (MLT), (**b**) PLA, (**c**) poly(ethylene adipate) (PEAd), and (**d**) poly(butylene adipate) (PBAd).

In that sense, we have recently reported the synthesis, full structural characterization, and unique physicochemical features of PLA and its copolymers, poly(ethylene adipate) (PEAd) and poly(butylene adipate) (PBAd) (Figure 1), used in this study [28,29]. The addition of a “softer” aliphatic polyester onto the macromolecular chain of PLA, i.e., PEAd or PBAd, was also found to severely affect the hydrolysis rate, thus leading to improved dissolution rates of the active pharmaceutical ingredients (API) [30,31].

In the present work, we utilized the aforementioned co-polyesters as co-formulants for the preparation of modified-release tablet formulations, to further investigate their potential in melatonin’s release. Their application was, indeed, proven to be of key importance, as MLT’s release was facilitated in all cases. As expected, the co-presence of HPMC K15, lactose monohydrate, and sodium alginate in the matrix tablets attenuated or augmented the MLT’s release, mainly depending on their respective content. To the best of our knowledge, this is the first time that these particular polymeric materials have been studied with respect to MLT’s oral delivery from matrix tablets.

## 2. Materials and Methods

### 2.1. Materials

Melatonin (Mw: 232,28, λ_max_: 278 nm) was purchased from Tokyo Chemical Industry (Tokyo, Japan). The new polymers (neat PLA, PLA/PEAd [90/10], PLA/PEAd [75/25], PLA/PBAd [90/10], and PLA/PBAd [75/25]) were kindly donated from the Laboratory of Professor Dimitrios Bikiaris, which is part of the Laboratory of Chemistry and Technology of Polymers and Dyes, in the Department of Chemistry of Aristotle University of Thessaloniki, Greece. HPMC K15M was supplied from Sigma-Aldrich (Steinheim, Germany). Alginic acid sodium salt (low viscosity) and Avicel PH 102 were obtained from Alfa Aesar GmbH & Co. KG (Karlsruhe, Germany). Lactose monohydrate was purchased from Merck (Darmstadt, Germany), whereas magnesium stearate was obtained from Riedel-De Haen (Hannover, Germany). All chemicals were of reagent grade and were used in the study without further purification.

### 2.2. Reclystallization of PLA, PLA-co-PEAd and PLA-co-PBAd

The requisite polymer, PLA, PLA-*co*-PEAd, and PLA-*co*-PBAd (1.45 g), in the form of large hard chunks, were sonicated with a mixture of ethyl acetate (25 mL) and dichloromethane (15 mL) at 35 °C for 30 min. The resulting clear solution was then treated dropwise with *n*-pentane at ambient temperature, until an off-white solid was precipitated. The solid was filtered in vacuo, washed with *n*-pentane (2 × 10 mL), and dried under vacuum in open air to quantitatively give the respective polymer, as a white powder, which was used in this form for the preparation of the matrix tablets.

### 2.3. Preparation of Melatonin Modified-Release Tablets

The matrix tablets of melatonin were prepared by blending and compressing with a variety of excipients (Table 1). The melatonin and excipients (copolymers of PLA, HPMC, sodium alginate, lactose monohydrate, and Avicel PH 102) were blended in a laboratory-scale powder blender at 32 rpm for 8 min (Wab Turbula type T2F). Afterwards, the lubricant, magnesium stearate, was added and mixing was continued for 2 more minutes. The powder mixture was accurately weighed (200 mg), loaded on a 10 mm diameter dye, and directly compressed using a hydraulic press (Maassen type, MP 150).

### 2.4. Tablet Uniformity Tests

The thickness of the tablets was measured using a Vernier caliper scale.

The hardness of the tablets was determined using an Erweka hardness tester (Erweka type TBH28). The force applied was equal to breaking the tablet in adiametric compression. The surface hardness of each tablet is expressed in N [32].

For the friability test, ten tablets were brushed to remove any overlying dust and were accurately weighed. These tablets were placed into the rotating drum of the friability test apparatus (Erweka type TA 3R, Heusenstamm, Germany). The drum was rotated at the speed of 25 rpm for 4 min. The tablets were de-dusted again and re-weighed. The percent friability was expressed by using the following equation:

(1) $$\mathrm{Friability}=\frac{\left(\mathrm{Initial}\;\mathrm{weight}-\mathrm{Final}\;\mathrm{weight}\right)}{\mathrm{Initial}\;\mathrm{weight}}\times 100\%$$

Uncoated compressed tablets that lost less than 1% (after 100 revolutions) of their weight were considered acceptable [32].

### 2.5. In Vitro Dissolution Studies

The in vitro dissolution tests were carried out in a dissolution test apparatus, USP type II (Pharmatest, Hainerp, Germany) (paddle method, 37 ± 0.5 °C, 50 rpm). The experiments were conducted in two different aqueous media; for the first 2 h, 450 mL of 0.2 M HCl solution (pH 1.2) was used to simulate the stomach pH, and to that, 450 mL of 0.14 M K_2_HPO_4_ solution (pH 9) was added to simulate the enteric pH (pH 6.8). Samples (5 mL) were withdrawn at predetermined time intervals, filtered, and analyzed in a Perkin–Elmer UV spectrophotometer (Norwalk, CT) at λ_max_ = 278 nm.

### 2.6. Methods to Compare Dissolution Profiles

Graphs of % MLT release vs. time were constructed, in order to compare the dissolution profiles.

The dissolution efficiency % [D.E. (%)] [33] value was calculated using the following equation:

(2) $$D.E.\;(\%)=\frac{\int_{t_{1}}^{t_{2}}\mathrm{ydt}}{y_{100}\left(t_{2}-t_{1}\right)}$$

where y is the percentage of dissolved MLT, and D.E. (%) is the area under the dissolution curve between time points t_1_ and t_2_, expressed as a percentage of the curve at maximum dissolution y_100_ over the same time period.

Additionally, the values referring to time, t_20%_, t_50%_ and t_90%_, in which 20%, 50% and 90% of MLT was released, were calculated.

The mean dissolution time (MDT) [34,35] values were calculated from the following equation:

(3) $$\mathrm{MDT}=\frac{\mathrm{ABC}}{W_{\infty }}$$

where W∞ is the maximum amount of MLT dissolved, and ABC is the area between the drug dissolution curve and its asymptote.

The in vitro release data were fitted to the Korsmeyer–Peppas equation to decipher the dissolution kinetics:

(4) $$\frac{M_{t}}{M_{\infty }}={\mathrm{kt}}^{n}$$

where M_t_ and M∞ refer to the absolute cumulative amount of drug released at time t and infinite time, respectively, with k as the release rate constant and n as the diffusion coefficient [36,37,38]. In the case of cylindrical tablets, n ≤ 0.45 denotes Fickian diffusion release (case I diffusional), 0.45 < n < 0.89 is non-Fickian anomalous transport, and n = 0.89 is zero-order (case II) release kinetics.

### 2.7. Attenuated Total Reflectance Infrared Spectroscopy (ATR-FTIR)

Attenuated total reflectance infrared spectroscopy (ATR-IR) was conducted using a Cary 670 infrared spectroscope (Agilent Technologies), fitted with a damping unit of total reflection with a diamond crystal (Accessories Attenuated Total Reflectance (ATR) diamond, model GladiATR, Pike technologies). The samples were screwed into position using the compression tip on the diamond accessory, and the spectra were collected with a resolution of 4 cm^−1^, from 4000 to 400 cm^−1^, as the sum of 32 scans.

### 2.8. X-ray Powder Diffraction (XRD)

X-ray powder diffraction (XRD) patterns of the analyzed samples were recorded using an XRD diffractometer (Rigaku, model MiniFlex II, Chalgrove, Oxford, UK) with CuKα radiation for crystalline phase identification (λ = 0.15405 nm for CuKα). The samples were scanned from 5 to 45°.

## 3. Results and Discussion

The results from the tablet uniformity tests were considered acceptable. In detail:

Thickness test: for all the formulations, the tablets’ diameter was 10 mm and the thickness was 2.8 ± 0.05 mm.

Hardness test: the results revealed that the tablets’ surface hardness was in the range of 55–100 N.

Friability test: the results from the friability test showed that the friability of the tablets was <1% in all cases, indicating adequate tablet strength.

**Figure 2:**
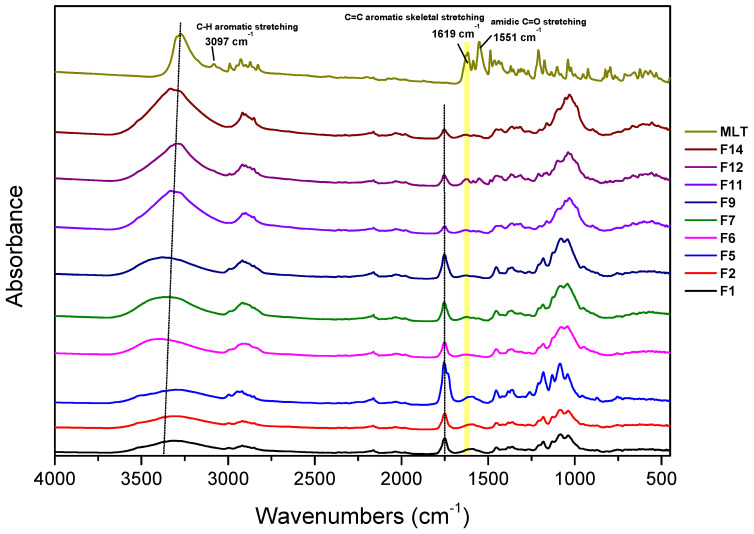
ATR-FTIR spectra of pure melatonin and selected prepared formulations (F1, F2, F5, F6, F7, F9, F11, F12, and F14).

An ATR-FTIR analysis was performed upon all formulations in an attempt to comprehend the drug–polymeric matrix interactions in the solid state. A typical ATR-FTIR spectrum collected from tablets contains a myriad of valuable information, hidden in a family of tiny peaks. ATR-FTIR characterization is complex, mainly due to the low amount of MLT added in the formulations (1 *w*/*w* %) and the high degree of overlapping absorption bands of the drug and the polymeric matrix [39]. Thus, emphasis was given to the most characteristic regions, corresponding to the C=O groups of the copolymers. In Figure 2, the ATR-FTIR spectra of pure melatonin and selected prepared formulations are depicted. Pure melatonin exhibits absorption bands at 3275 cm^−1^, 3097 cm^−1^, 1619 cm^−1^, and 1211 cm^−1^, which correspond to the N-H stretching vibration, the C-H aromatic stretching, the C=C aromatic skeletal stretching vibration, and the C-O-C (C5-OCH_3_) stretching vibration, respectively. In addition, the C=O stretching of the amidic carbonyl group of melatonin is observed at 1551 cm^−1^ [40,41,42].

**Figure 3:**
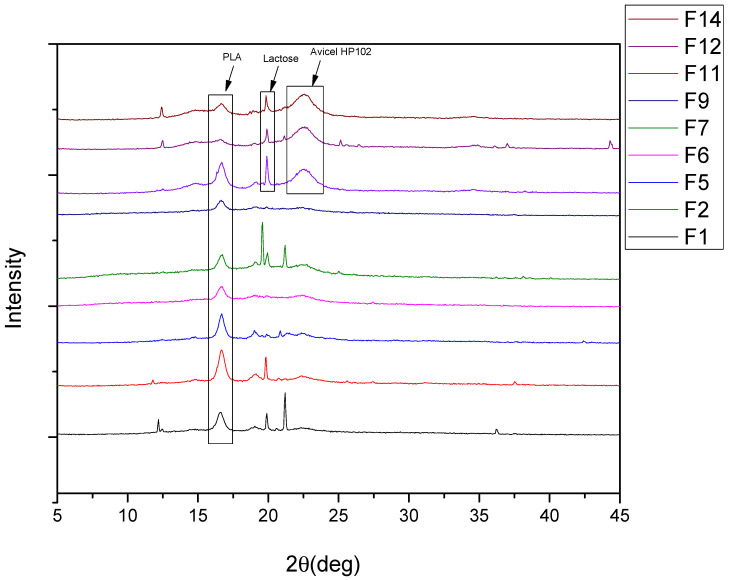
XRD pattern of the prepared tablet formulations.

X-ray diffraction (XRD) was employed in order to determine the crystallinity of the melatonin formulations. It is shown that melatonin exhibits sharp peaks at the diffraction angles of 2θ = 16.3, 24.2, 25.0, and 26.0°, indicating its crystalline structure (Figure S3, Supplementary Section) [42,43]. Pure melatonin is a monoclinic crystal, and the typical peaks in the range 2θ 10–30° denote the long-range order of its supramolecular structure [44]. Furthermore, in all the formulations’ XRD patterns, the characteristic peaks of melatonin do not appear, suggesting that MLT is well dispersed inside the tablets and has an amorphous form. The dissolution of drugs is significantly enhanced by amorphization, and, thus, is highly desired in drug delivery applications, thereby denoting the therapeutic potential of MLT formulations [45,46]. Generally, in (semi)crystalline/(semi)crystalline polymer blends, the peak intensities recorded in the XRD patterns depend on the concentration of each polymer, as different components crystallize separately [47]. From Figure 3, it is observed that the peaks, corresponding to each polymeric blend, are present, with the most noticeable at 16.8°, being attributed to PLA. Special allusion should be made to the F11, F12, and F14 formulations. Specifically, the narrow peak exhibited at 19.9° corresponds to the presence of lactose, while the wide peak at 22.5° is attributed to the increased amount of Avicel PH 102.

**Figure 4:**
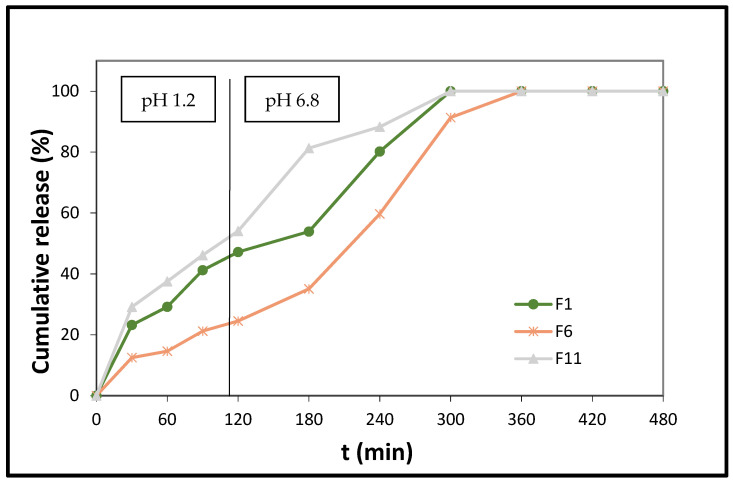
In vitro % release of melatonin from formulations F1, F6, and F11 vs. time. The results represent the mean value (n = 3, SD < 2).

It becomes apparent from the results presented in Figure 4 that the release of melatonin from the polylactic acid-based (PLA) formulation F1 reaches 47.20% at t = 120 min, and is completed at t = 300 min. The facile release of melatonin, at the acidic medium, is possibly due to the fact that, although the PLA’s carboxyl groups, present in the F6 tablets, remain undissociated, its free OHs, via H-bond formation with the C5-methoxyl and C3-ethanamido groups of melatonin, enhance the solubilization. Regarding the interactions between the drug and polymeric matrix, it has been previously reported, by Pandey et al. [48], that melatonin interacts well with polymeric matrixes, such as PLA. In our case, considerable interaction is noticed in the area of the carbonyl group of the copolymers, where peaks from 1755 cm^−1^ shift towards lower wavenumbers in all the formulations, as a result of the intermolecular hydrogen bond formation between the C=O of PLA and the *Ν*1-H and -NH amido groups (in the non-charged resonance form) of MLT (Figure 2) [48,49,50].

The influence on melatonin’s release of the relevant quantity of HPMC K15 in the PLA-containing tablets is as expected, since the release of the hormone from the matrices, where HPMC K15 is present in high quantity (F6), is much lower than that from the respective low-quantity HPMC K15-containing F11 tablets. It is well known that HPMCs, in general, when in contact with aqueous media, form gel layers, which delay the penetration process of water molecules into the matrix structure [51,52,53].

The lower and slower release of MLT from the F6 formulation, with respect to formulation F1, can also be attributed to the fact that, in F1, the amount of lactose monohydrate is two-fold higher (F6: 8 mg vs. F6: 16 mg). Lactose, being a water-soluble substance, leads to faster polymer chain relaxation, thus facilitating the API’s diffusion from the hydrophilic polymeric matrix [53]. This difference in the pace of MLT’s release is even more profound in the case of the F11 formulation. In the F11 matrix tablets, the content of lactose monohydrate is even higher than in F6 (F11: 20 mg vs. F6: 8 mg). In addition, because the amount of sodium alginate in the F11 tablets is more than half of that present in formulation F6, the release of MLT from the former tablets is 54.07% at t = 120 min, while, from the latter, it is 24.51% at t = 120 min. Sodium alginate, at the acidic dissolution medium (0 min ≤ t ≤ 120 min), does not exist as a salt (it is converted to alginic acid), and, as a result, the lower its quantity in the matrix systems, the higher the release of the API [54]. Moreover, MLT’s release is enhanced in the F11 tablets, compared to F1 and F6, because of the increased amount of Avicel PH 102 in the F11 matrices (Figure S4, Supplementary Material; the wide peak at 22.4° in the diffractogram is attributed to Avicel PH 102); besides contributing to compactional strength and rapid disintegration, it facilitates the release of the drug [55].

The release of MLT from these three formulations renders them suitable for undisturbed sleep maintenance. Furthermore, the formulations F1 and F11 are also suitable for dealing with sleep-onset problems.

In general, it is well known that the composition of the copolymer can directly affect several of the physicochemical material properties, such as hydrophilicity, structure, morphology, and, most importantly in our case, the drug–polymer interactions. As an overall observation for all the studied copolymer-based formulations, it is evident that the release rate of MLT proceeds more slowly at pH 1.2 than at pH 6.8. This trend can be explained as follows: when the polymer-based formulated tablet is in contact with the buffer medium, the copolymer becomes hydrated and subsequently swells. The drug molecules, physically entrapped within the matrix, are then also in contact with the buffer. As a result of this hydration process, the existing drug–polymer physical interactions are lowered, and the drug can more easily diffuse out of the swelled polymer. Now, in the acidic medium (pH 1.2), the hydrophilic parts of the copolymer (PEAd or PBAd units) remain protonated, thus restricting the formation of extensive H-bonding with water and leading to reduced swelling ability, which, in turn, leads to slower diffusion of the drug molecule and, therefore, slower release rates.

**Figure 5:**
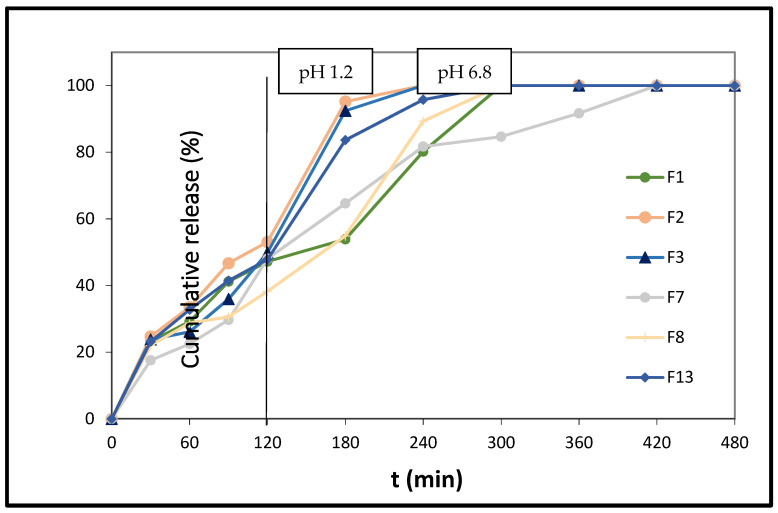
In vitro % release of melatonin from formulations F1, F2, F3, F7, F8, and F13 vs. time. The results represent the mean value (n = 3, SD < 2).

In the case of poly(ethylene adipate) (PEAd), the relevant content in the PLA matrix tablets (F2, F3, and F13; Figure 5) does not seem to play an important role in the release of melatonin from the respective formulations. In the former two cases, the release of the hormone is completed at t = 240 min, and it follows the same pace. In the case of MLT’s release from the F13 formulation, the release becomes quantitative at t = 300 min. This relatively fast release could be attributed to the presence of free OHs and ester groups in the PEAd structure, which act synergistically with the free OHs of PLA in the H-bond formation between them and the C5 and C3 functionalities of the melatonin nucleus. It is noteworthy that upon reduction of the amount of PLA/PEAd ([90/10], 33.5 mg) in half in the F7 matrix tablets, compared to their F2 congeners (PLA/PEAd [90/10], 67 mg), MLT’s release becomes substantially lower after t = 120 min, reaching 100% at t = 420 min. An analogous effect is noticed in the case of the F13 formulation. It seems that in these cases, the above H-bond interactions are minimized, as the amount of substrate (PEAd) is drastically reduced. These arguments are also corroborated by the ATR-FTIR spectral data of the tablet formulations, showing the existence of wide peaks at 3000–3600 cm^−1^, due to the combined presence of the -NH groups of MLT, the -OH groups deriving from cellulosic excipients, as well as any remaining free -OH from the copolymers used in the tablets (Figure 2). The considerable interaction noticed in the area of the carbonyl group of the copolymers, where peaks from 1755 cm^−1^ shift towards lower wavenumbers in all the formulations, results, in this case, from the intermolecular hydrogen bond formation between the *Ν*1-H and -NH amido groups (in the non-charged mesomeric form) of the drug, and the carbonyl groups of the copolymers. Moreover, the widening of the peak at 1619 cm^−1^ of the C=C aromatic skeletal stretching is also attributed to possible drug–polymer interactions [48].

**Figure 6:**
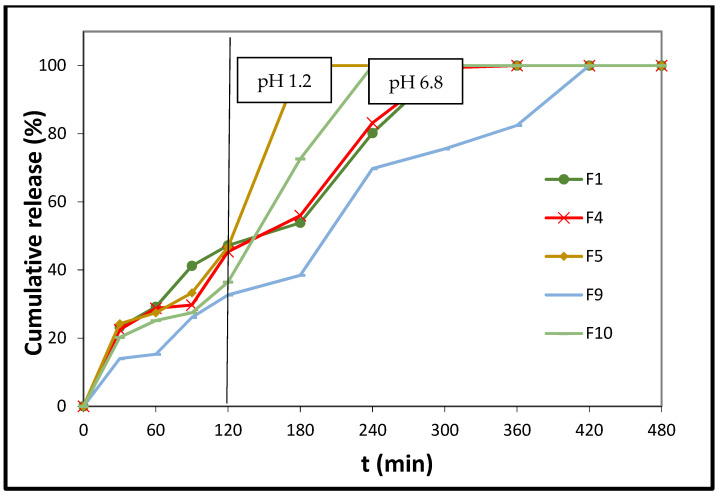
In vitro % release of melatonin from formulations F1, F4, F5, F9, and F10 vs. time. The results represent the mean value (n = 3, SD < 2).

In the case of poly(butylene adipate) (PBAd)/PLA matrix systems, the relevant content of PBAd (F4 and F5, F9 and F10; Figure 6) plays a more important role in the release profile of melatonin. Thus, the higher the content of PBAd in the tablets (F5), the higher and faster the release of the hormone (F5: 100% release at t = 180 min vs. F4: 100% release at t = 300 min). An analogous trend is observed in the cases of the F9 and F10 tablets. A plausible explanation for this would be the increased number of PBAd ester groups in the F5 and F10 cases, with respect to the number of respective ester groups in the F4 and F9 matrix tablets.

**Figure 7:**
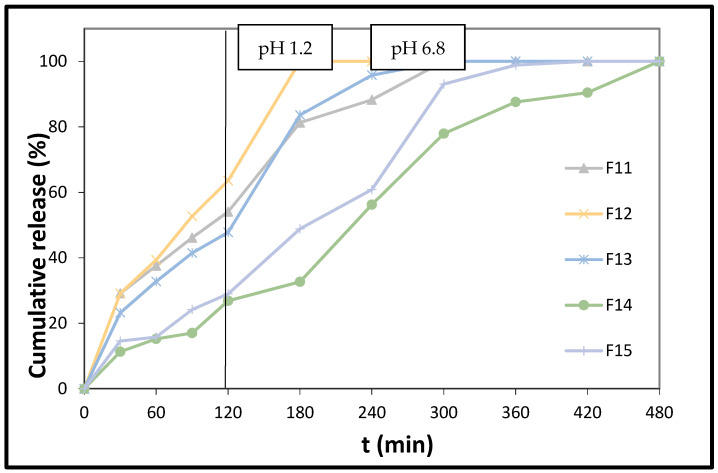
In vitro % release of melatonin from formulations F11, F12, F13, F14, and F15 vs. time. The results represent the mean value (n = 3, SD < 2).

However, the difference in the release of the hormone from (PBAd)/PLA tablets, where PBAd is present in low (F14; Figure 7) and high amounts (F15; Figure 7), and, concurrently, the amount of sodium alginate is very low (7 mg), is negligible.

Conversely, the difference in MLT’s release, noticed between the PBAd/PLA-containing tablets (F14) and those including PEAd/PLA (F12), is significant, and has to do with the discrete crystallization of these polyesters within the polymeric blend. The PLA/PEAd blends showed characteristic peaks at 2 theta 17.5, 20.65, 21.7, and 24.86 deg of PEAd. The PBAd/PLA blends exhibited a peak at 2 theta 21.9°, which is the characteristic peak corresponding to PBAd (Figure 3: XRD pattern of PEAd/PLA 90/10 and XRD pattern of PBAd/PLA 90/10).

As in the cases of F1 and F11, the release of MLT from the formulations F2, F3, F11, and, especially, F5 renders them suitable for dealing with sleep-onset problems. Conversely, the pace of release of the hormone from F14 and F15 better suits the requisite profile for sleep maintenance.

The kinetic release properties of the developed formulations are reported in Table 2. In particular, the kinetics data revealed that MLT’s release followed, in most cases, [F1 (n = 0.49), F2 (n = 0.58), F3 (n = 0.63), F4 (n = 0.52), F5 (n = 0.52), F9 (n = 0.65), F11 (n = 0.46), F12 (n = 0.60), and F13 (n = 0.53)], anomalous diffusion, whilst MLT’s release from formulation F7 followed zero-order kinetics (n = 0.89).

## 4. Conclusions

The results presented herein accurately demonstrate the subtle differences in the in vitro modified release of melatonin, upon variation in the composition and relevant content of PLA, PLA-*co*-PEAd, and PLA-*co*-PBAd (95/5, 90/10, 75/25 and 50/50 weight ratios) in the developed matrix tablets. Indicatively, the release of MLT from the formulations F6, F7, F8, F26, and, especially, F10 renders them suitable for dealing with sleep-onset problems. Conversely, the pace of release of the hormone from the F29 and F30 matrix tablets better suits the requisite profile for sleep maintenance. It is possible that these observations are due to predominant H-bonding interactions between the drug and copolymers, whereas the swelling ability of the copolymers may have an impact on the release rates. The latter should be further investigated in the future. The fact that the difference in copolymer composition plays a role in MLT’s release rate demonstrates its potential for tuning the hormone’s delivery. We are currently pursuing analogous studies on the release of melatonin, using, as formulants, a variety of different block copolymers.

## Figures and Tables

**Table 1:** Composition of melatonin tablet formulations.

Ingredients	F1	F2	F3	F4	F5	F6	F7	F8	F9	F10	F11	F12	F13	F14	F15
Melatonin	2	2	2	2	2	2	2	2	2	2	2	2	2	2	2
Neat PLA	68					34					34				
PLA/PEAd [90/10]		68					34					34			
PLA/PEAd [75/25]			68					34					34		
PLA/PBAd [90/10]				68					34					34	
PLA/PBAd [75/25]					68					34					34
HPMC K15	16	16	16	16	16	120	120	120	120	119.5	16	16	16	16	16
Sod.Alginate	78	78	78	78	78	16	16	16	16	16	8	8	8	8	8
Lactose	16	16	16	16	16	8	8	8	8	8	20	20	20	20	20
Avicel PH 102	20	20	20	20	20	20	20	20	20	20	120	120	120	120	120
Mg.Stearate	2	2	2	2	2	2	2	2	2	2	2	2	2	2	2
Total	200	200	200	200	200	200	200	200	200	200	200	200	200	200	200

**Table 2:** Kinetic release properties of the developed formulations.

Formulations	MDT	t_20%_	t_50%_	t_90%_	n	Mean % D.E.
F1	170.24	24	140	270	0.49	70.06
F2	127.55	22	102	177	0.58	78.90
F3	136.36	22	120	179	0.63	77.10
F4	142.20	24	142	262	0.52	69.67
F5	133.48	22	152	169	0.52	77.68
F6	192.80	81	218	299	1.21	59.44
F7	151.35	41	122	341	0.89	67.40
F8	171.82	24	162	240	0.38	69.77
F9	193.70	72	201	384	0.65	58.11
F10	158.14	30	141	219	0.42	72.67
F11	140.08	20	102	245	0.46	76.16
F12	115.05	20	82	162	0.60	81.37
F13	141.63	24	122	210	0.53	76.02
F14	208.44	100	222	400	1.09	54.25
F15	179.30	78	180	296	0.98	62.12

## Data Availability

Not applicable.

## Supplementary Materials

The following supporting information can be downloaded at: https://www.mdpi.com/article/10.3390/polym14081504/s1, Figure S1: FT-IR spectra of the depicted excipients used in the prepared formulations; Figure S2: FTIR spectra of neat PLA, PEAd, PBAd, PLA/PBAd (90/10), the (75/25) copolymers and the PLA/PEAd (90/10), (75/25) copolymers; Figure S3: XRD pattern of pure melatonin; Figure S4: XRD patterns of (a) Avicel PH 102, (b) HPMC K15, (c) sodium alginate, (d) lactose, and (e) magnesium stearate; Figure S5: XRD pattern of neat PLA before and after recrystallisation; Figure S6: XRD patterns of the studied (a) PLA-PEAd, and (b) PLA-PBAd copolymers.
