# Supplementary material for: Modified Release of the Pineal Hormone Melatonin from Matrix Tablets Containing Poly(L-lactic Acid) and Its PLA-co-PEAd and PLA-co-PBAd Copolymers
Source: Polymers (Basel). 2022 Apr 7;14(8):1504. doi: 10.3390/polym14081504 (PMC9027688; doi:10.3390/polym14081504)
Supplement: Supplementary file 1 [file polymers-14-01504-s001.zip › polymers-1653976-supplementary.pdf]

polymers

SUPPLEMENTARY INFORMATION

Modified Release of the Pineal Hormone Melatonin From Matrix Tablets Containing Poly(*L*-lactic Acid) and Its PLA-*co*-PEAd and PLA-*co*-PBAd Copolymers

Marilena Vlachou [1,*], Angeliki Siamidi [1], Dionysia Anagnostopoulou [1], Evi Christodoulou [2] and Nikolaos D. Bikiaris [2]

[1] Division of Pharmaceutical Technology, Department of Pharmacy, School of Health Sciences, National and Kapodistrian University of Athens, Panepistimioupoli-Zografou, 15784 Athens, Greece; asiamidi@pharm.uoa.gr (A.S.); dionanag@pharm.uoa.gr (D.A.)
[2] Laboratory of Polymer Chemistry and Technology, Department of Chemistry, Aristotle University of Thessaloniki, 54124 Thessaloniki, Greece; evicius@gmail.com (E.C.); nbikiaris@gmail.com (N.D.B.)
* Correspondence: vlachou@pharm.uoa.gr; Tel.: +302107274674

1. Attenuated Total Reflectance-Infrared Spectroscopy (Atr-Ftir)

Figure S1 shows the characteristic peaks observed for all the biopolymers used as excipients in the fabricated formulations.

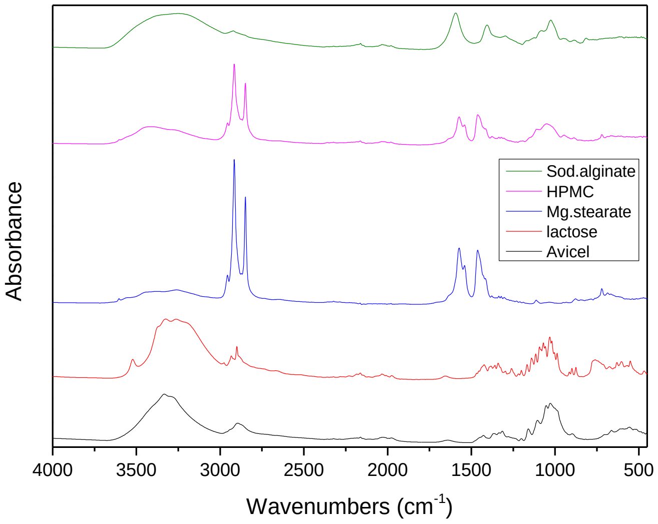

Figure S1. FT-IR spectra of the depicted excipients used in the prepared formulations.

In the FTIR spectrum of sodium alginate, stretching vibrations of O–H bonds of alginate were observed in the range of 3000–3600 cm^{-1}. The absorption peak at 2921 cm^{-1} is attributed to the stretching vibrations of aliphatic C–H. The observed bands at 1594 and 1406 cm^{-1} are due to the asymmetric and symmetric stretching vibrations of the carboxylate salt ion, respectively. Later bands are very significant and can be used for characterization of the alginate structure from its derivatives and ingredients. The bands at 1080 and 1025 cm^{-1} are derived from the C–O stretching vibration of the pyranosyl ring and the C–O stretching with contributions from the C–C-C and C-O–H deformations [1].

Regarding the spectrum of lactose monohydrate, the absorption bands observed at 3600–3200 cm^{-1} are characteristic of the stretching vibrations of the lactose alcohol groups existing either as free or bonded. The two sharp bands at 2896 and 2933 cm^{-1} are been as-

signed to the stretching vibrations of two types of C–H bonds: those that were within the constituents of lactose (glucose and galactose units), and those of the methyl alcohol group outside the glucose and galactose units. The weak peak shown at 1653 cm^{-1} corresponds to the stretching vibrations of water from the crystallization of the OH bonds and of water adsorbed on the surface of the lactose. The bands observed at 1500–1200 cm^{-1} also describe the bending vibrations of the C–H bonds. All bands occurring between 984 and 1204 cm^{-1} are attributed to the asymmetrical stretching vibrations of the C–O–C ether unit bond [2].

The spectrum of Avicel PH 102 exhibited an O-H stretching absorption peak at 3332 cm^{-1}, a C-H stretching absorption at 2894 cm^{-1}, and a C-O-C stretching peaks around 900 and 1170 cm^{-1}. These absorption peaks are in accordance with those of the typical cellulose backbone [3].

With respect to the HPMC K15 spectrum, strong bonded hydroxyl bands were observed at 3050-3600 cm^{-1} and two sharp bands at 2845 and 2917 cm^{-1}, which correspond to the C–H alkyl groups stretching. In the 1120–1020 cm^{-1} region the observed absorption bands are attributed to the (C-O-C) group of cellulose [4].

Last, the twin peaks at 1572 and 1461 cm^{-1}, observed in the magnesium's stearate spectrum, are attributed to the asymmetric carboxylate (COO-) and symmetric carboxylate stretching vibrations, respectively, whereas the absorption peaks at 2915 and 2849 cm^{-1} correspond to the stretching vibration of the C–H group [5].

Figure S2 depicts the FT-IR spectra of neat PLA as well as those of the prepared PLA/PEAd, PLA/PBAd block copolymers. PLA exhibits asymmetric and symmetric vibrations of the methylene groups at 2995 cm^{-1} and 2945 cm^{-1}, respectively, while the vibrations of the carbonyl C=O and the C-O-C ester groups are recorded at 1757–1710 cm^{-1} and 1188 cm^{-1}. The spectra of neat PEAd exhibited a characteristic absorption peak at 1738 cm^{-1} attributed to the carbonyl group, while the 3315–3700 cm^{-1} area and the peak at 2961 cm^{-1} at the O-H and C-H groups, respectively. In the case of PBAd, the characteristic absorption peaks of the ester -COO- and the C-O-C appeared at 1735 cm^{-1} and 1100–1300 cm^{-1}, respectively, while the peaks observed at 1450–1465 cm^{-1} correspond to the C-H bending vibrations of the methylene and methyl groups. In all spectra the low intensity peaks recorded at 3300–3550 cm^{-1} can be attributed to the presence of the -OH end groups.

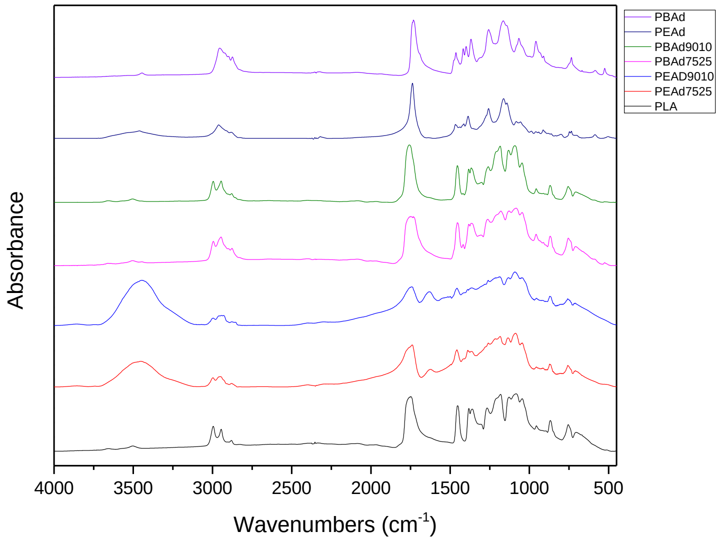

Figure S2. FTIR spectra of neat PLA, PEAd**,** PBAd, PLA/PBAd (90/10), the (75/25) copolymers and the PLA/PEAd (90/10), (75/25) copolymers.

Regarding the spectra of the synthesized PLA/PEAd copolymers, a double peak in the copolymers is recorded in the carbonyl region around 1750 cm^{-1}. The peak at lower wavenumbers correspond to carbonyl of the adipic acid groups in PEAd, while the peak located at higher wavenumbers corresponds to the PLA carbonyl groups, thus confirming the existence of both polymers in the final materials. Finally, intensity absorptions occurring in the range of 3600–3300 cm^{-1} could only be associated with O-H bonds, either from unreacted ethylene glycol from the PEAd molecules or from the terminal hydroxyls in the molecules. In the target materials these absorptions are increased, which may indicate side-reactions during polymerization of materials and formation of O-H bonds. Regarding the recorded spectra of the PLA/PBAd copolymers, the results revealed increased similarities to each other. Specifically, in all cases a strong absorption peak at 1730 cm^{-1} was recorded, due to the formation of a new ester bond between the PLA and the PBAd (accountable for the formation of the block copolymer). Moreover, several peaks in the areas of 750–1100 cm^{-1} and 1100–1400 cm^{-1}, correspond to the C-C and C-O vibrations, respectively. Finally, the presence of more methylene groups, within all synthesized copolymers, was also confirmed by the increase of the intensities of the specific peaks recorded in the 2700–3000 cm^{-1} region [6].

2. X-Ray Powder Diffraction (XRD)

According to the obtained XRD patterns (Figure S4), both HPMC K15 and sodium alginate were found to exist in amorphous form, and thus they did not exhibit any characteristic peaks linked with the crystalline shape. On the other hand, Avicel PH 102 gave characteristic crystalline peaks at 2θ= 14.7, 22.4 and 33.6°, lactose at 2θ=19.0,19.9 and 39.9°, whereas in the case of magnesium stearate three crystalline peaks were observed at 2θ=21.8, 22.5 and 23.4°.

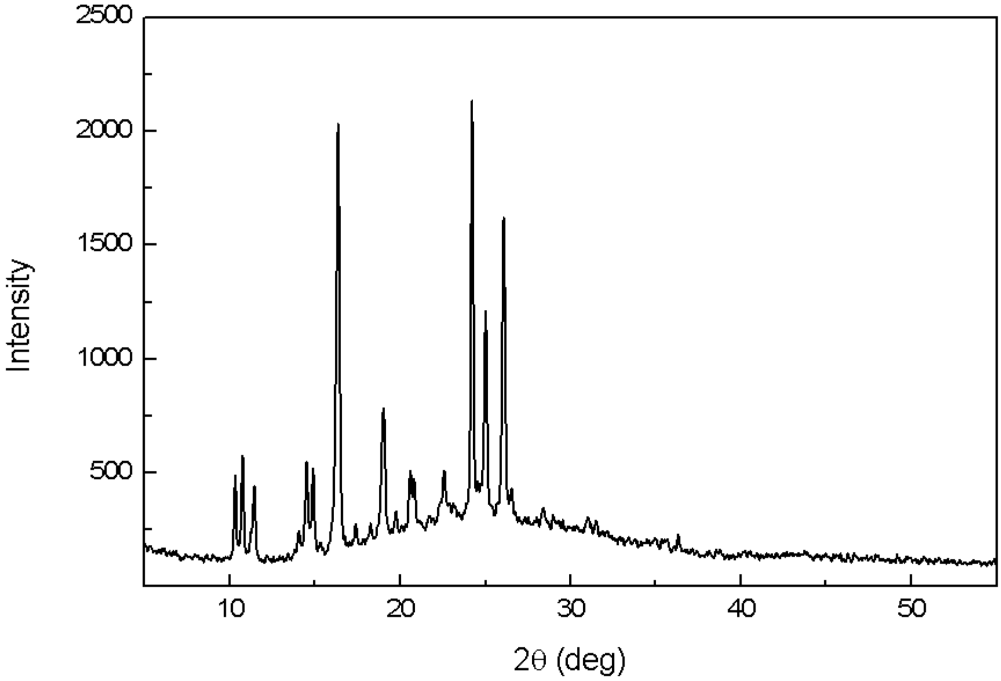

Figure S3. XRD pattern of pure melatonin.

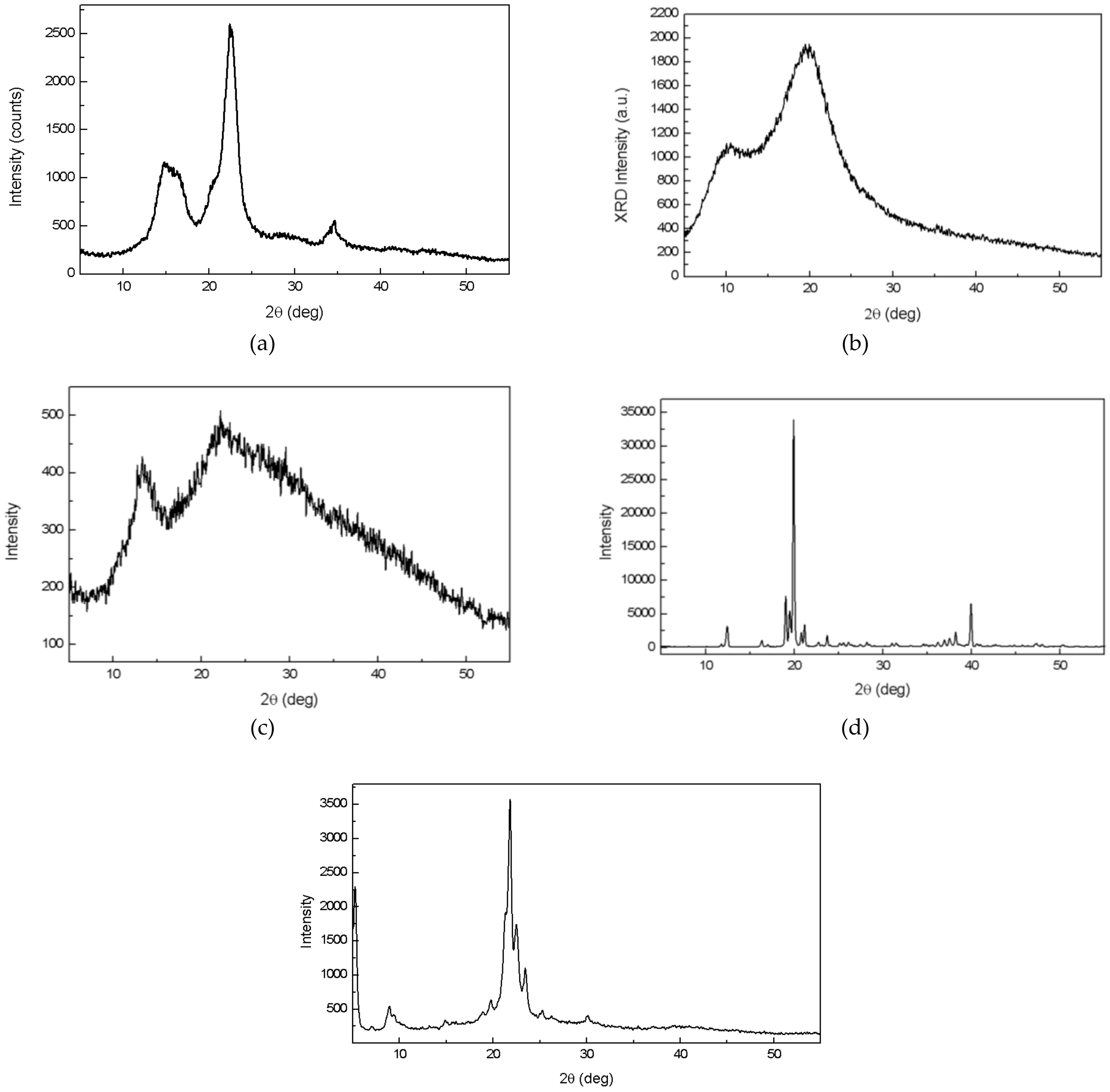

(e)

Figure S4. XRD patterns of (a) Avicel PH 102, (b) HPMC K15, (c) sodium alginate, (d) lactose, and (e) magnesium stearate.

With respect to PLA, the measuremnt was recorded at room temperature (RT) after it had previously been melted and fully annealed at 115 °C. After recrystallization it exhibited characteristic peaks at 14.9,16.8,19.1 and 22.4° (Figure S5).

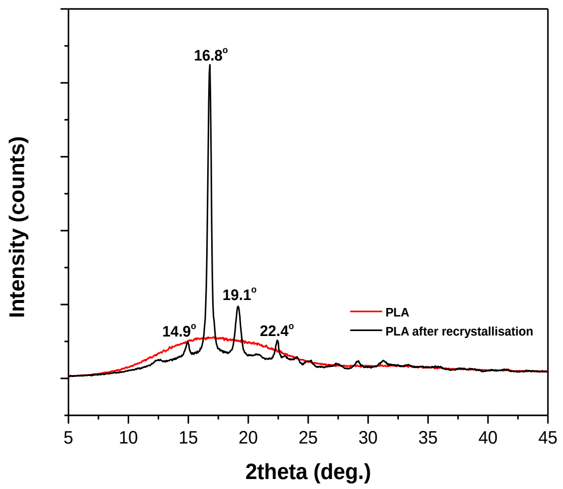

Figure S5. XRD pattern of neat PLA before and after recrystallisation.

When examining the XRD patterns of PLA/PEAd and PLA/PBAd polymeric blends, the corresponding diffraction peaks of both neat polyesters were recorded in all cases (Figure S6), while the peak intensities were dependent on the concentration of each polymer, suggesting that the prepared polyesters were immiscible and crystallize separately within the polymeric blend. All polymeric blends exhibited semi-crystalline structure. PLA/PEAd blends showed the characteristic peaks at 2 theta 17.5, 20.65, 21.7, and 24.86 deg of PEAd. Moreover, the PLA/PBAd blends exhibited at 2 theta, 21.9° the characteristic peak corresponding to PBAd.

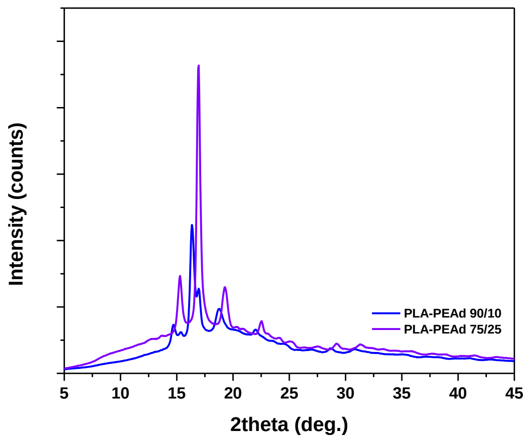

(a)

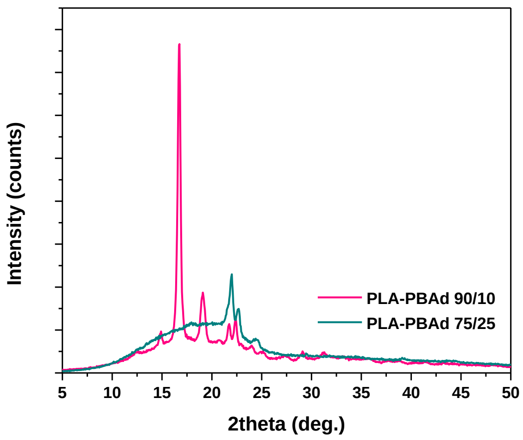

(b)

Figure S6. XRD patterns of the studied (a) PLA-PEAd, and (b) PLA-PBAd copolymers.

References

1. Daemi, H.; Barikani, M. Synthesis and characterization of calcium alginate nanoparticles, sodium homopolymannuronate salt and its calcium nanoparticles. *Scientia Iranica*, **2012**, *19*, 2023–2028.
2. Listiohadi, Y.; Hourigan, J.A.; Sleigh, R.W.; Steele, R.J. Thermal analysis of amorphous lactose and alpha-lactose monohydrate. *Dairy Science and Technology*, **2009**, *89*, 43–67.
3. Setu, M.N.I.; Mia, M.Y.; Lubna, N.J.; Chowdhury, A.A. Preparation of microcrystalline cellulose from cotton and its evaluation as direct compressible excipient in the formulation of Naproxen tablets. *Dhaka University Journal of Pharmaceutical Sciences*, **2014**, *13*, 187–192.
4. Bianchi, S.E.; Angeli, V.W.; Souza, K.C.B.D.; Miron, D.D.S.; Carvalho, G.D.A.; Santos, V.D.; Brandalise, R.N. Evaluation of the solubility of the HPMC: PVA blends in biological fluids in vitro. *Materials Research*, **2011**, *14*, 166–171.
5. Nep, E.I.; Conway, B.R. Preformulation studies on grewia gum as a formulation excipient. *Journal of Thermal Analysis and Calorimetry*, **2011**, *108*, 197–205. https://doi.org/10.1007/s10973-011-1782-4.
6. Karava, V.; Siamidi, A.; Vlachou, M.; Christodoulou, E; Bikiaris, N.D.; Zamboulis, A,; Kostoglou, M.; Gounari, E.; Barmpalexis, P. Poly(l-Lactic Acid)-co-poly(Butylene Adipate) New Block Copolymers for the Preparation of Drug-Loaded Long Acting Injectable Microparticles. *Pharmaceutics*, **2021**, *13*, 930.
